# The mode of action of Fruitless: Is it an easy matter to switch the sex?

**DOI:** 10.1111/gbb.12606

**Published:** 2019-09-05

**Authors:** Kosei Sato, Daisuke Yamamoto

**Affiliations:** ^1^ Neuro‐Network Evolution Project, Advanced ICT Research Institute National Institute of Information and Communications Technology Kobe Japan

**Keywords:** axon guidance, chromatin remodeling, courtship behavior, *Drosophila*, neuritegenesis, RNA polymerase II, sexually dimorphic neurons, the *fruitless* gene, transcription factor, ubiquitin‐proteasome pathway

## Abstract

The *fruitless* (*fru*) locus was originally defined by a male sterile mutation that promotes male‐to‐male courtship while suppressing male‐to‐female courtship in *Drosophila melanogaster*. The *fru* promoter‐1 pre‐RNA generates a set of BTB‐zinc finger family FruM proteins expressed exclusively in the male neurons, leading to the formation of sexual dimorphisms in neurons via male‐specific neuroblast proliferation, male‐specific neural survival, male‐specific neuritegenesis or male‐specific arbor patterning. Such a wide spectrum of phenotypic effects seems to result from chromatin modifications, in which FruBM recruits Bonus, Histone deacetylase 1 (HDAC1) and/or Heterochromatin protein 1a (HP1a) to ~130 target sites. One established FruBM transcriptional target is the axon guidance protein gene *robo1*. Multiple transcriptional regulator‐binding sites are nested around the FruBM‐binding site, and mediate sophisticated modulation of the repressor activity of FruBM. FruBM also binds to the Lola‐Q transcriptional repressor to protect it from proteasome‐dependent degradation in male but not female neurons as FruBM exists only in male neurons, leading to the formation of sexually dimorphic neural structures. These findings shed light on the multilayered network of transcription regulation orchestrated by the master regulator FruBM.

## Graphical abstract

*Drosophila* Fruitless regulates proteolysis to produce sex‐specific Lola isoforms that specify neuronal sex types.

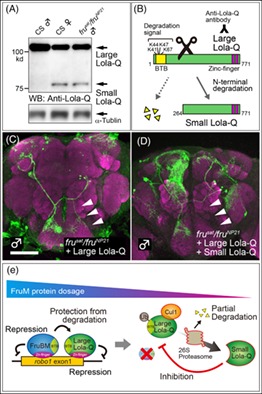

## 1 FRUITLESS AS THE PRIMARY ORGANIZER OF MALE COURTSHIP CIRCUITS

**Figure 1:**
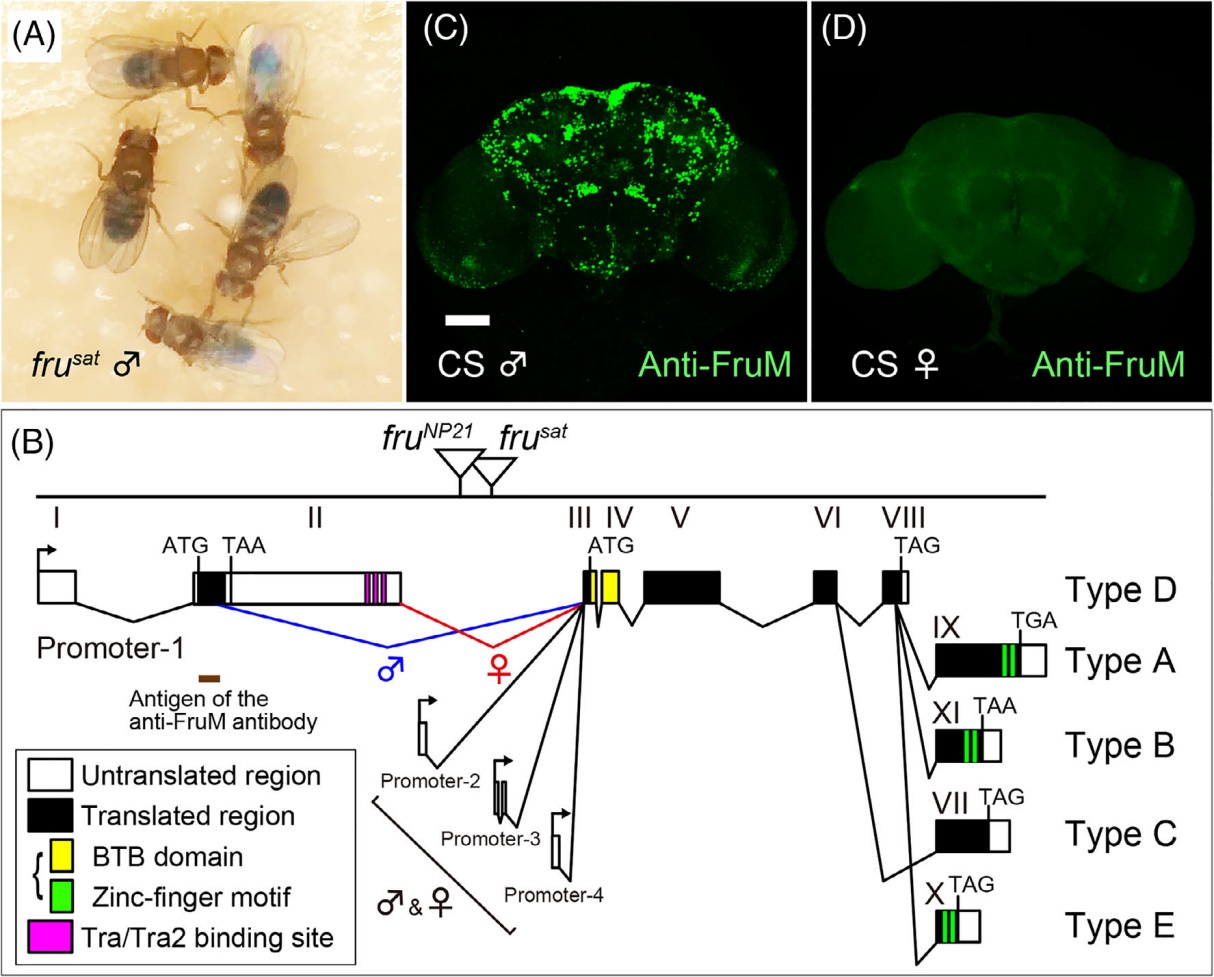
Behavioral phenotype, gene structure and protein expression for the *fruitless* locus. A, *fru* mutant (*fru* ^*sat*^) males courting each other. B, The *fru* locus. The P‐element insertion sites for *fru* ^*NP21*^ and *fru* ^*sat*^ alleles (upper) and the exon (exons I‐XI)—intron organization (lower) for five isoforms (types A‐E) are schematically illustrated. Start and stop codons are also indicated. C and D, Anti‐FruM antibody staining shows FruM expression in the male (C) but not female (D) brain from the wild‐type strain Canton‐special (CS) flies. Note that the terminology of Fru isoforms used in this article—originally introduced by Usui‐Aoki et al.^5^—is different from that used by S. F. Goodwin's group and B. J. Dickson's group—originally introduced by Song et al.^28^: our FruA, B and E correspond to their FruA, FruC and FruB, respectively. Scale bar: 50 μm

**Figure 2:**
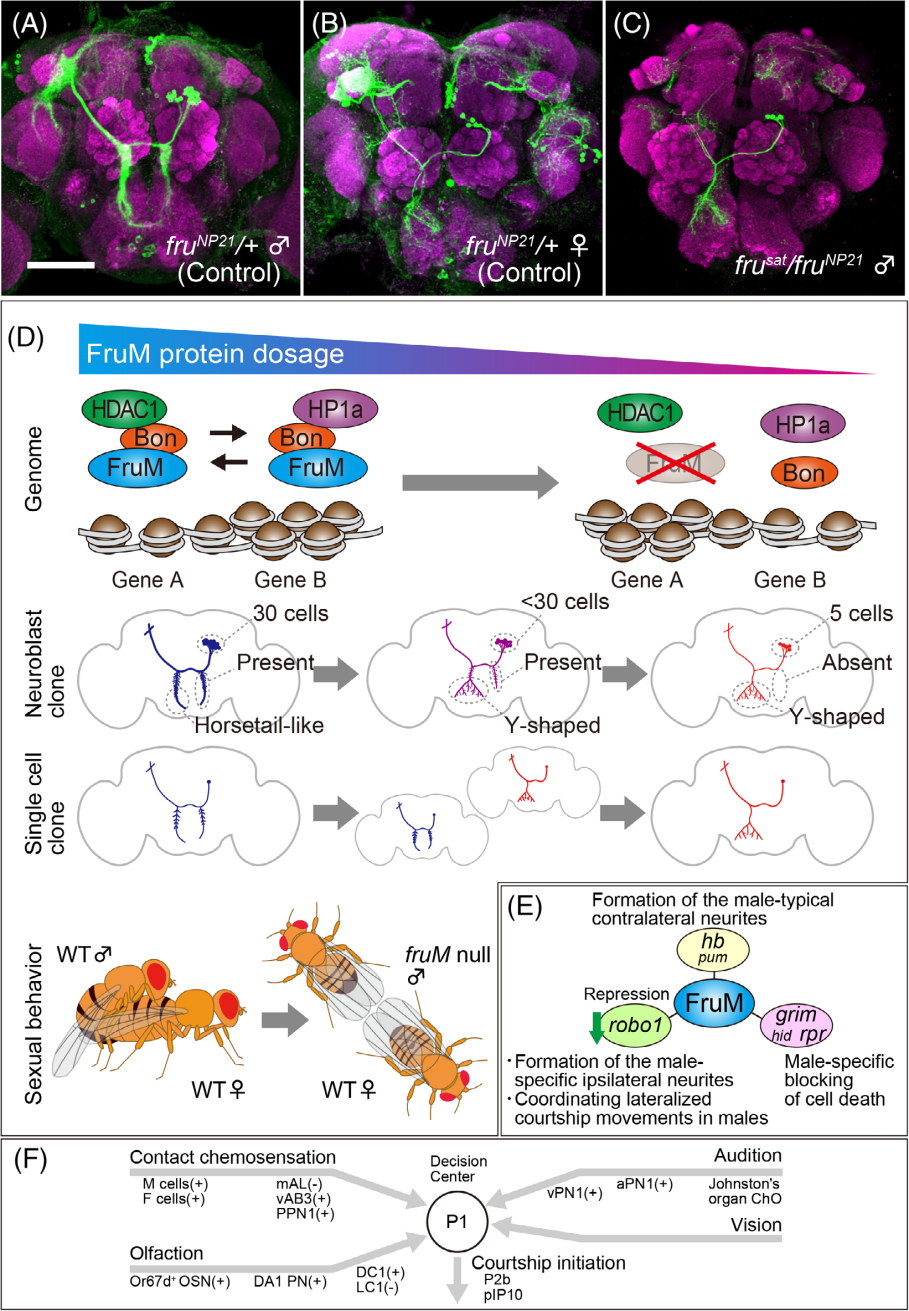
Cellular phenotypes induced by loss of FruM and triads of FruM functions. A, Images of mAL neurons in a normal (*fru* ^*NP21*^ heterozygous) male (A) and female (B), and a *fru* heteroallelic mutant (*fru* ^*sat*^ */fru* ^*NP21*^) male (C). D, Hypothetical FruM action as a chromatin regulator. The model postulates that chromatin relaxes as the FruM amount reduces (upper) in mutant males and concomitant changes in gene expression result in an increase in the proportion of female‐type mAL neurons at the expense of male‐type neurons (middle), accompanied by a reduction in courtship activities (lower). When all mAL neurons are visualized in toto in *fru* hypomorphic males, superposition of the male‐type and female‐type neurons results in an intersexual appearance of the entire mAL neural cluster (neuroblast clone *cf*. single cell clone in the middle panel). Three aspects of mAL sex differences, that is, differences in the cell number, the branching pattern of the contralateral neurite and the presence or absence of the ipsilateral neurite are also indicated on schematic drawings of mAL neurons in the middle panel. E, Three aspects of sex differences are all regulated by FruM yet via distinct downstream target genes. F, Simplified diagram of input and output pathways for P1 neurons. (+) and (−) indicate excitatory and inhibitory inputs, respectively. Scale bar: 50 μm

The fruitfly *Drosophila melanogaster* offers an outstanding opportunity to unravel the molecular and cellular underpinnings of complex traits, including sociosexual relations among conspecifics, due to its rich genetic resources for manipulation and its relatively simple circuitries composed of a smaller number of neurons. The neural pathway for courtship behavior represents one of the best characterized circuitries in the fly. This is primarily due to the discovery of a courtship‐defective mutant, *fruitless* (*fru*; see Figure 1A for the behavioral phenotype of *fru* ^*sat*^),^1^ and the subsequent identification of the gene responsible for it, that is, the *fru* gene (Figure 1B), which produces a set of proteins key to the courtship circuitry formation in male flies.^2^, ^3^ There are ~2000 *fru*‐positive neurons in the adult nervous system,^4^, ^5^ which collectively represent roughly 2% of the entire neuron population (Figure 1C,D). Many of the *fru*‐positive cells exhibit sex differences in structure and/or number, and some of them are only present in either sex.^6^, ^7^, ^8^, ^9^, ^10^, ^11^ Loss of *fru* converts the cellular sex type in some neurons, often accompanying phenotypes in sex‐related behaviors (Figure 2A‐C). By using such changes in behavioral outputs as readouts of sexual transformation in the neurons involved, one can identify the neurons that play a pivotal role in the production of a given behavior (Figure 2D,E). With the aim of determining the cells that induce male‐type courtship behavior in a female fly, Kimura et al.^8^ sexually transformed a small number of neurons in the female brain with the aid of a clonal technique, MARCM.^12^ This attempt identified a male‐specific neural cluster, called P1, composed of 20 *fru*‐expressing interneurons per hemisphere (the P1 neurons also express another sex determinant gene, *doublesex*).^8^ Thermogenetic or optogenetic activation of the P1 cluster drives a solitary male to initiate courtship, and the P1 neurons increase intracellular Ca^2+^ (signifying an excited state) when exposed to a live female or dummy targets with appropriate properties as courtship inducers.^13^, ^14^, ^15^, ^16^, ^17^ These findings led to the proposition that the P1 cluster functions as the center that makes the decision of whether or not to mate (Figure 2F). Subsequent studies have confirmed and extended this seminal observation by various approaches. The neurons that convey inputs reporting sensory cues to the P1 cluster as well as putative pathways operating downstream of the P1 cluster for motor control have been identified: e.g., inhibitory mAL and excitatory vAB3 and PPN1 as direct presynaptic interneurons for P1,^18^, ^19^, ^20^ and P2b and pIP10 as direct postsynaptic interneurons.^15^, ^21^, ^22^ These interneurons connecting with P1 neurons turned out to be *fru*‐positive, reinforcing the idea that the core portion of the courtship neural circuit is built up by connections among *fru*‐positive neurons.^23^ The question arises as to how *fru* orchestrates the formation of mutual connections between a specific set of *fru*‐positive neurons to organize the entire courtship circuit. To answer this question, we need to know the molecular modes of action of *fru* gene products.

## 2 FRUITLESS AS A TRANSCRIPTION FACTOR

The *fru* gene displays an exceedingly complex organization (Figure 1B). It has at least four promoters; the most distal promoter *Promoter‐1* is dedicated to sex‐related functions, while others are used to generate non‐sex‐specific Fru proteins FruCOM.^3^, ^5^, ^24^, ^25^ The *Promoter‐1*‐derived primary transcript is processed differently between females and males, depending on the presence or absence of Transformer (Tra), a female‐specific splicing regulator known to function as a feminizer protein in the sex‐determination cascade.^26^ In females, Tra binds to the *fru* primary transcript at sites locating distally in exon‐2, resulting in a long mRNA that includes the exon‐2 sequence containing a translation stop signal; Figure 1B). In males, Tra is not expressed and thus the *fru* primary transcript is spliced at a more proximal site, yielding a shorter mRNA that lacks the exon‐2‐derived stop codon. As a result, *fru* mRNA in males can encode a large open reading frame for proteins (FruM; M stands for male) whereas that in females is unable to encode a protein.^3^, ^5^ An obvious outcome of this alternative splicing is the production of proteins that are male‐specific^4^, ^5^, ^27^ (Figure 1C,D). It remains an open question whether the noncoding female transcript has any biological activity. Alternative splicing also occurs non‐sex‐specifically near the 3′ end of the *fru* primary transcript, producing C‐terminal variants referred to as FruAM, FruBM, FruCM, FruDM and FruEM (FruM members), or their non‐sex‐specific counterparts (FruCOM members) FruA, FruB, FruC, FruD and FruE, at least in theory (some of these proteins have not been experimentally detected).^28^ Although *fru* isoform‐specific mutants have been generated, the functional differences of these proteins in neural sex differentiation were not fully elucidated.^29^, ^30^ The absence of any of the specific FruM isoforms leads to defects in male sexual behavior, but in a different strength and pattern. For example, FruBM‐less males completely fail to copulate, FruEM‐less males are less successful in mating, and FruAM‐less males exhibit almost normal mating activities, except for anomalies in certain courtship song parameters. Although most *fru*‐positive neurons express all three of these isoforms together, some neurons express only two or one isoform(s). It remains to be clarified whether the function or the expression of the isoforms is responsible for the observed phenotypic differences among isoform‐specific mutants.

## 3 ALL‐OR‐NONE SEX SWITCHING BY CHROMATIN REMODELING

At the molecular level, the FruAM, FruBM and FruEM isoforms have been characterized in some detail. FruM, but not FruCOM, has an N‐terminal extension of 101 a.a., which is immediately followed by a common broad‐complex, tramtrack, and bric‐a‐bric (BTB) domain, whereas the C‐terminus has two zinc finger motifs unique to each isoform^3^, ^5^ (Figure 1B). The BTB domain is known to function as a protein dimerization interface, and the zinc finger motif mediates DNA binding, suggesting that FruM may form a transcription factor complex. In support of this proposition, FruBM binds to ~130 sites on the polytene chromosome as showed by immunostaining with an anti‐Fru antibody.^31^ Most of these chromosomal sites were also positive for Bonus (Bon), a transcriptional cofactor homologous to mammalian TIF1 as well as histone deacetylase 1 (HDAC1), and 20 out of 100 Fru‐bound sites (some of them overlap the HDAC1‐positive sites) were labeled with an antibody that recognizes the heterochromatin protein 1a (HP1a). Bon was dispensable for FruM binding to the genomic sites, but HDAC1 and HP1a fail to bind to the FruM‐positive sites in the absence of Bon, implying that FruM binds the site first, Bon binds the site second and then Bon recruits HDAC1 and/or HP1a.^31^ A complementary experiment to immunoprecipitate these proteins substantiated the hypothesis that FruM, Bon, HDAC1 and/or HP1a form a complex on these specific sites on the genome. The likely scenario is that a FruM‐containing complex switches chromatin states, thereby silencing or activating a large set of genes that are required in neurons for taking on the female or male fate (Figure 2D). Behaviorally, *bon* and *HDAC1* (*Rdg3*) mutations agonistically act with *fru* mutations in abrogating male courtship activities, whereas *HP1a* (*Su(var)205*) mutations negatively modulate the effects of *fru* mutations. Possible effects of these mutations on sex‐specific neural development were examined by clonally labeling the mAL cluster of *fru*‐positive neurons, because this particular cluster displays conspicuous sex differences: the number of cells composing the cluster is 5 in females and 30 in males; the ipsilateral neurite is present in some male neurons but totally absent in all female neurons; the tip of the contralateral neurite in the subesophageal ganglion always bifurcates in females whereas it has a comb‐like appearance without a bifurcation in males^7^ (Figure 2D). All these sexual characteristics of mAL neurons were completely feminized in loss‐of‐function *fru* mutant males (*Promoter‐1* null mutants).^7^ A single cell clone analysis unequivocally showed that reductions in *fru* or *HDAC1* gene activity increased the female‐type mAL neurons, whereas similar reductions in *HP1a* gene activity in *fru* hypomorphic males increased male‐type mAL neurons. A remarkable fact is that no single mAL neuron with intersexual characteristics was found in these genetic variants.^31^ All individual neurons examined had a perfect female‐type or perfect male‐type structure; it was only the proportion of female‐type and male‐type neurons that changed in these flies (Figure 2D). Thus, FruM seems to act as a switch in a binary choice of sexual fate determination of neurons. However, potential FruM downstream genes have impact on a select aspect of the sexual dimorphism in mAL neurons (Figure 2E): loss of three major cell death‐inducing genes, *grim*, *hid* and *reaper*, affects primarily the sex difference in the number of mAL neurons^7^; the transcription factor Hunchback selectively acts to shape the sexually‐dimorphic branching of the contralateral neurite^32^; *robo1* (see below) specifically affects the sex‐dependent presence or absence of the ipsilateral neurite.^33^ mAL neurons represent probably the most conspicuous example of neural sexual dimorphism, but many other *fru*‐positive neurons are also sexually dimorphic, as showed by the dedicated efforts to resolve the finer structures of individual neurons on the standardized brain. Some neural clusters are only found in the female or male brain as a result of sex‐specific cell death or sexually distinct proliferation patterns of a neuroblast. Thus, the mechanistic triad underlying the sexual dimorphisms found in mAL neurons would also be useful in understanding the mechanisms for sex‐type specification in other *fru*‐positive neurons.

## 4 DIRECT TRANSCRIPTIONAL TARGET OF FRUITLESS

**Figure 3:**
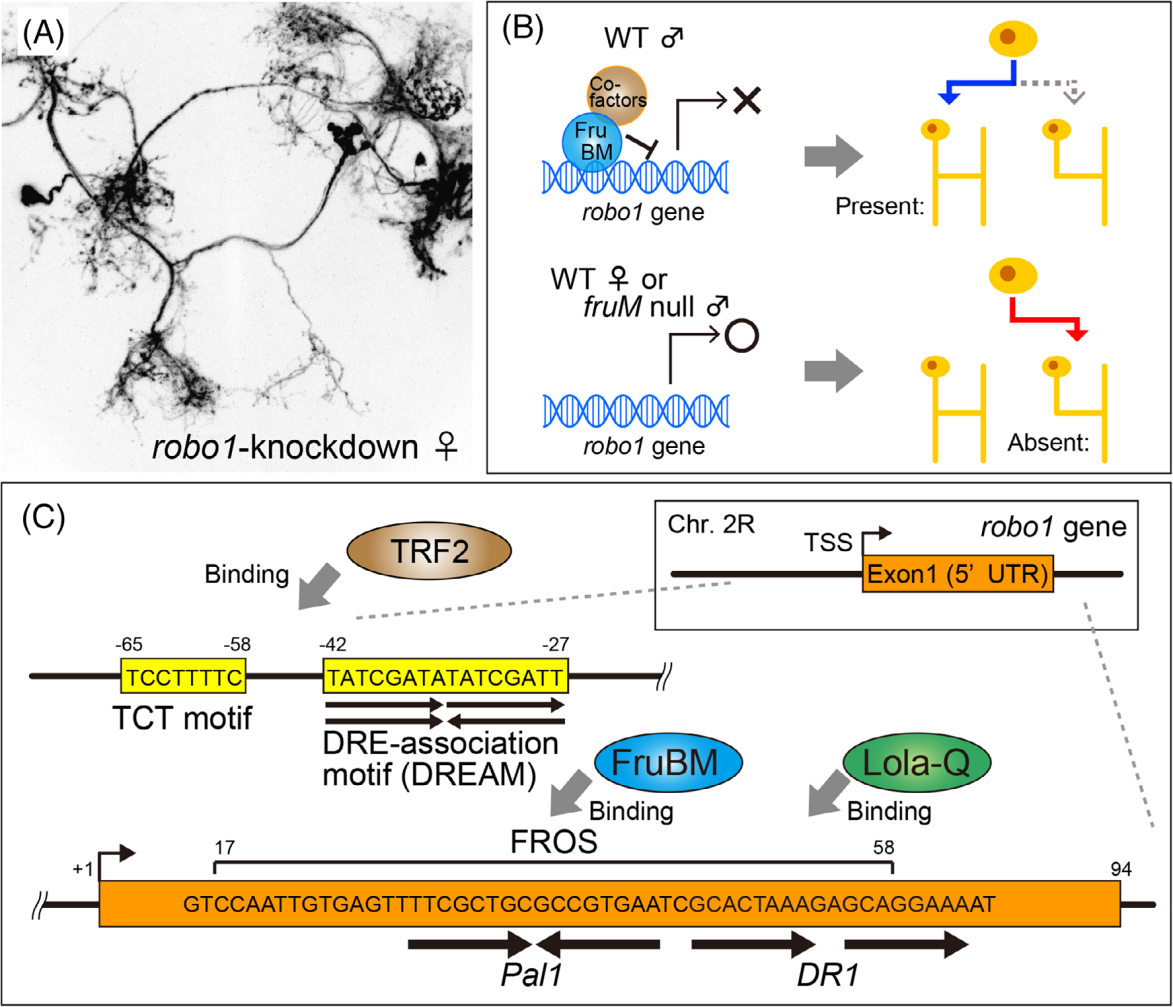
The FruBM target *robo1*. A, *robo1* knockdown results in the male‐specific neurite formation in female mAL neurons. B, Schematic drawing of the mechanism whereby FruBM regulates the male‐specific neurite formation in mAL neurons. C, FruBM, TRF2 and Lola‐Q each interact with distinct motifs around the *robo1* transcriptional start site (TSS) to coordinately regulate *robo1* transcription

Which genes are transcriptionally regulated by FruM? Vernes^34^ conducted immunoprecipitation assays coupled to deep sequencing (ChIP‐seq) with lysates from *fru*‐transfected cultured S2 cells, and a complementary reverse transcription PCR (RT‐PCR) experiment with RNAs extracted from male flies. In this attempt, ~100 potential targets for each of the major three Fru isoforms were obtained; she found that ion channel‐encoding genes were the dominant group, biases existed for X‐chromosome‐linked genes and increased transcription was prevalent rather than downregulation upon Fru overexpression. Dalton et al.^35^ inferred binding consensus sequences for Fru isoforms by screening synthetic DNAs with random sequences for their ability to bind to in vitro translated Fru proteins. Neville et al.^29^ employed the DamID technique: a bacterial DNA methylase sequence (Dam) fused to FruM is transgenically expressed in *fru*‐expressing neurons in vivo, so that the FruM moiety binds to a target DNA sequence, which in turn is methylated by the Dam moiety and thus tagged.^36^ This approach allowed them to deduce likely binding sequences for FruM isoforms. Expression analysis combined with *fru* knockdown for tens of genes locating close to the deduced FruM‐binding sequences on the genome led them to propose that *CadN* and *lola* are two most likely candidates for FruEM targets.^29^ Meissner et al.^37^ found that expression of the GAL4 reporter knocked into the *Leucine‐rich repeat G‐protein coupled receptor 3* (*Lgr3*) gene is more intense in females than males, and this sexual dimorphism in *Lgr3* reporter expression disappears when FruEM is knocked down. They identified several possible FruEM‐binding sites in an *Lgr3* intron by electromobility shift assays (EMSA) in vitro; however, mutating all these sites in the GAL4 knock‐in allele failed to affect the sexually dimorphic *Lgr3* expression in vivo,^37^ making it difficult to conclude that *Lgr3* is a direct target for FruEM. Dauwalder et al.^38^ screened for sex‐specific transcripts in the brain, and identified one from the *takeout* gene that encodes a putative lipid transporter expressed in the fat body associated with the brain. Although both *fru* and *dsx* stimulate transcription of the *takeout* gene, it is unknown whether they coordinately act on the *takeout* regulatory sequence. We cannot exclude other possibilities; for instance, *fru* and *dsx* might regulate each‐others' transcription, thereby affecting expression of their target effector genes, such as *takeout*. Ito et al.^33^ attempted a phenotype‐based candidate gene approach to identify FruBM transcriptional targets: a collection of “neural genes” was knocked down in *fru*‐expressing mAL neurons to obtain ones that convert the neural sex‐type, with a particular focus on the presence or absence of the male‐specific ipsilateral neurite upon knockdown. This search yielded *robo1*, a gene encoding a transmembrane receptor for the neurite guidance signal Slit^39^, ^40^; *robo1* knockdown in females resulted in an ectopic formation of the male‐specific contralateral neurite in females yet without effect in males^33^ (Figure 3A). This result implies that Robo1 normally inhibits the ipsilateral neurite formation in females, whereas this Robo1‐dependent inhibition is removed in males, allowing them to develop the ipsilateral neurite in mAL neurons (Figure 3B). Ito et al.^33^ found that it is FruBM that removes Robo1 from male mAL neurons: FruBM binds to the *robo1* promoter to repress its transcription. Gel‐mobility shift and reporter assays showed that the FruBM binding requires a 42 bp region (named FROS) in the *robo1* promoter encompassing a palindrome sequence, TTCGCTGCGCCGTGAA (named *Pal1*; Figure 3C).^33^ *Pal1* contains the motif proposed as a FruM binding sequence by Neville et al.^29^ but does not contain any motif proposed by Dalton et al.^35^ Ito et al.^33^ discovered that a small deletion of a several base pair region in *Pal1* of the *robo1* promoter (eg, a 6 bp deletion occurring in the *robo1* ^*Δ1*^ allele) not only increased the number of male flies with no male‐specific neurite but also impaired the male courtship posture, even in the flies carrying only one copy of the mutation, that is, heterozygotes. When they generate courtship songs, normal males vibrate only one wing at a time, and they alternate the wing to vibrate once every several seconds. However, males carrying *robo1* ^*Δ1*^ alternate the wing to vibrate several times per second (the precocious wing‐switching phenotype). This observation suggests that leaky expression of *robo1* occurs if FruM fails to bind to the *robo1* promoter, with the consequence that both the neurons and behavior are de‐masculinized, and these changes manifest as dominant phenotypes as in the case of the *robo1* ^*Δ1*^ heterozygotes.

## 5 FRUBM REPRESSOR ACTIVITY MODULATED BY TRF2

The *robo1* gene promoter offers an unparalleled opportunity to dissect the molecular mechanism by which the actions of FruM lead to the sex‐specification of individual neurons and ultimately to sex‐specific behavior. In fact, the *robo1* promoter harbors additional motifs for binding of other transcription factors around the FruM‐binding site FROS. Located ~30 nt upstream of the *robo1* transcription start site (corresponding to ~50 nt proximal to the FROS) is the DNA‐replication element association motif (DREAM), which is proximally flanked by the TCT motif (Figure 3C). Genetic screens for phenotypic *fru* modifiers identified TATA‐box‐binding protein‐related factor 2 (TRF2),^41^ which is known as a transcriptional activator that preferentially binds to the core promoter sequence with a TCT motif.^42^, ^43^ DNA replication‐related element‐binding factor (DREF), a known binding partner of TRF2, binds to DRE,^44^ whose palindrome repeat is contained in a DREAM motif. As expected, the *robo1* promoter fragment with TCT and DREAM motifs binds TRF2. What is interesting, however, is the result of the reporter assay with the *robo1* promoter fragment in transfected S2 cells: TRF2 activates transcription when applied singly, but when applied together with a low concentration of FruBM that is insufficient for transcription repression, TRF2 actually represses transcription—that is, TRF2 now potentiates the FruM repressor action.^41^ This finding raises the intriguing possibility that local interactions of FruBM with TRF2 on the *robo1* promoter might underlie the context‐dependent switching between activation and repression of *robo1* transcription.

## 6 FRUITLESS AS A SEX‐SPECIFIC PROTEASOME REGULATOR

**Figure 4:**
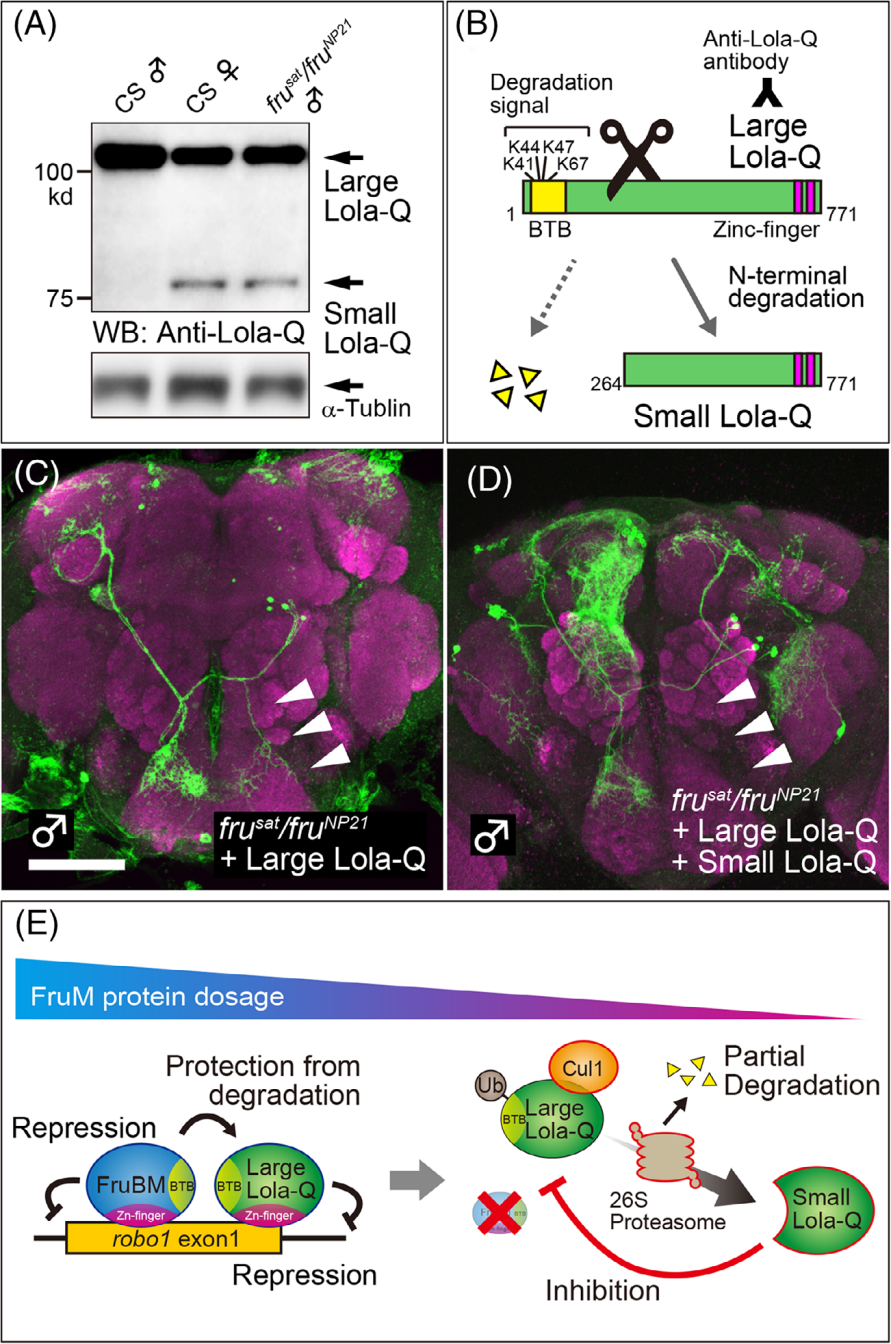
The second mode of action of FruBM in the control of neural sex differentiation. A, Western blot analysis (WB) with the anti‐Lola‐Q antibody (for its epitope location, see panel B) shows that small Lola‐Q is a female‐specific form while large Lola‐Q is shared by the two sexes. *fru*‐mutant males express female‐specific Lola‐Q. B, Small Lola‐Q is produced from large Lola‐Q by partial proteasomal degradation. C and D, Overexpression of large Lola‐Q rescues the male‐specific neurite (arrowhead) in a *fru* mutant mAL cluster (C), which otherwise lacks it (see Figure 2c), and additional overexpression of small Lola‐Q counteracts this large Lola‐Q‐dependent rescue (D). E, FruBM interacts with large Lola‐Q on the *robo1* promoter to protect large Lola‐Q from degradation, and thereby repressing *robo1* transcription (left). As the amount of FruBM decreases, large Lola‐Q transforms into small Lola‐Q via proteasomal degradation, whereby restoring *robo1* transcription through inhibition of the large Lola‐Q action

In addition to TRF2, *fru* genetic modifier screens repeatedly identified *lola*,^32^ which encodes an exceedingly large number of isoforms of the BTB‐zinc finger protein family,^45^, ^46^ to which Fru also belongs. Because many BTB‐zinc finger proteins interact via each other's BTB domain,^47^ it is tempting to postulate that FruBM and Lola form a complex to regulate transcription of their shared target genes. Sato et al.^48^ discovered that isoform‐Q is composed of two distinct molecules with different molecular weights, that is, small Lola‐Q enriched in females and large Lola‐Q with male‐biased expression (Figure 4A,B). Large Lola‐Q represses the *robo1* reporter transcription in S2 cells, whereas small Lola‐Q counteracts large Lola‐Q. EMSA assays identified tandem direct repeats of 18 nt (named *DR1*) as the Lola‐Q‐binding site, which are mostly included within FROS flanking 3′ to the *Pal1* palindrome, that is, the core binding motif for FruBM (Figure 3C). Reporter assays in S2 cells showed a synergistic action between large Lola‐Q and FruBM in repressing transcription from the *robo1* promoter.^48^ Consistent with such synergy at the molecular level, overexpression of large Lola‐Q mitigates cellular phenotypes of *fru* loss‐of‐function; e.g., it restores the male‐specific neurite otherwise lost in *fru* mutant males (Figure 4C). This activity of large Lola‐Q to rescue the male‐specific neurite in *fru* mutant males is abrogated by simultaneous overexpression of small Lola‐Q (Figure 4D), in keeping with the inhibitory effect of small Lola‐Q on large Lola‐Q at the molecular level (see above). These observations suggest that female‐specific small Lola‐Q is indeed a feminizing factor, whereas large Lola‐Q with male‐biased expression is a masculinizing factor of *fru*‐positive sexually dimorphic neurons.^48^ At the behavioral level, deletion of *DR1* in the *robo1* promoter (*robo1* ^*Δ4*^; shown in Sato et al.^48^) induces precocious wing‐switching during male courtship reminiscent of the phenotype in flies that carry *robo1* ^*Δ1*^.^33^

Peptide sequencing by Edman degradation showed that small Lola‐Q lacks N‐terminal amino acid residues 1‐263 of large Lola‐Q, including the entire BTB domain.^48^ No 5′ variants are detected in mRNAs encoding Lola‐Q by 5′RACE, and therefore, the two forms of Lola‐Q likely result from posttranslational modification. Of note, the BTB domain is known to anchor ubiquitin proteasome components,^49^, ^50^ and thus a likely scenario is that large Lola‐Q is partially degraded to produce small Lola‐Q by ubiquitin proteasomes. Substitution of N‐terminal lysine residues that offer potential ubiquitination sites in flies or administration of proteasome inhibitors in S2 cells protects the conversion of large Lola‐Q into small Lola‐Q, supporting the idea that N‐terminal truncation of Lola‐Q involves ubiquitin proteasomes.^48^ Although ubiquitin‐dependent proteasomal processing typically brings about the complete degradation of substrates, there are cases where it produces truncated substrates with biological activities; some of the key signaling molecules are, in fact, such truncation products, including NFκB, yeast transcription factors Spt23 and Mga2, *Drosophila* Ci and its mammalian homolog Gli3, Epstein‐Barr virus‐protein EBNA1, and the RNA polymerase degradation factor Def1 in yeast. Interestingly, mass spectrometric analysis of immunoprecipitates with an anti‐Lola‐Q antibody identified Cullin1, an E3 ligase that polyubiquitinates lysine residues, which are in turn targeted by the 26S proteasome for degradation. Knockdown of either *Cullin1* or *proteasome subunit beta 5* induced the male‐specific neurite in some mAL neurons, in accord with the hypothesis that the masculinizer large Lola‐Q is converted into the feminizer small Lola‐Q by partial proteasomal degradation in female, but not male, flies^48^ (Figure 4E). Then, why does this degradation reaction take place only in females? The key to this sex‐specificity is the presence or absence of FruBM. FruBM is only present in males, where it binds to large Lola‐Q via the BTB‐BTB interaction, protecting large Lola‐Q from degradation by proteasome enzymes. Females are devoid of FruBM, and as a consequence, large Lola‐Q is processed to small Lola‐Q by proteasomal degradation.^48^ Thus, FruBM controls transcription in two ways: first, by changing chromatin states via the recruitment of a chromatin regulator complex containing Bon, HDAC1 and/or HP1a, and second, by turning on and off the activity of the repressor complex by regulating partial degradation of Lola‐Q in the complex.

## 7 UNSOLVED ISSUES

The fly brain contains ~2000 *fru*‐positive neurons, forming approximately 50 clusters of somata each uniquely locating within the brain.^4^ Every cell within a cluster appears to have a unique arborization and thus unique connections with other neurons, even if cells comprising the same cluster are typically alike. For example, the 29 male mAL neurons registered in NBLAST are classified into four groups based on their neurite branching patterns, such that rigorous characterization of the fine branching might allow one to identify a single neuron in a cluster.^51^ We cannot exclude the possibility that two neurons exhibiting a subtle structural difference within a cluster participate in distinct connections and thus distinct behavior, rather than forming redundant connections contributing to the improvement of safety factors of the system. How these subtle differences in the neurite pattern are produced are elusive. At best, we are just beginning to understand how gross differences in neuronal structures between the sexes are produced under the control of the master regulator gene *fru*. The *robo1* promoter has served as a useful model in exploring the molecular bases for FruBM actions in the sex‐type specification of neurons. Based on the picture emerging from the investigation of FruBM actions on the *robo1* promoter, we may need to reconsider the traditional view of the “master regulator” in a given developmental process. Turning ON and OFF middle management genes encoding transcription factors is not the sole job with which FruBM is charged. For example, FruBM also fine tunes the mode of action of a transcription complex to which it contributes, a complex that binds to the DNA motif on terminal effector genes encoding structural proteins. Strikingly, the FruBM core‐binding motif *Pal1* in the *robo1* promoter is surrounded by multiple putative binding motifs for different transcriptional regulators, two of which were identified as those for TRF2 and Lola‐Q. While TRF2 and Lola‐Q can bind to their own binding motifs on the *robo1* promoter, both proteins are included in the FruBM‐containing protein complex. This could mean that the FruBM‐containing protein complex may induce altered folding of the DNA strand around the binding sites. As salivary chromosome immunostaining unraveled ~130 FruM‐bound sites distributed across the chromosomes,^31^ one can envisage that similar folding may occur all across the genome, possibly generating higher‐order folding through the association of juxtaposed folding structures. Dense assemblies of transcriptional machinery called super‐enhancers (SEs) are produced to ensure the robust expression of genes, and such clustering of enhancers is mediated by transcriptional cofactors, for example, BRD4 and MED1, which form biomolecular condensates visible as coactivator puncta in the nucleus.^52^ Although FruBM appears to contribute more often to repressor complexes than activation complexes, cooperative assembly of regulatory elements analogous to SE might underlie FruBM‐mediated transcriptional control. The formation of SEs likely involves liquid‐liquid phase separation, which requires intrinsically disordered regions (IDRs) of BRD4 and MED1 to occur.^52^ FruBM seems to have a large IDR and is often localized in nuclear puncta (our preliminary observations). Structural‐biological analysis of FruBM is thus an essential approach toward further clarification of the means by which FruBM orchestrates transcription of multiple genes for the neural sex‐type specification.
